# Using species distribution modeling to generate relative abundance information in socio‐politically unstable territories: Conservation of Felidae in the central‐western region of Mexico

**DOI:** 10.1002/ece3.10534

**Published:** 2023-09-17

**Authors:** Juan F. Charre‐Medellín, David Ferrer‐Ferrando, Tiberio C. Monterrubio‐Rico, Javier Fernández‐López, Pelayo Acevedo

**Affiliations:** ^1^ National School of Higher Studies Universidad Nacional Autónoma de México Morelia Mexico; ^2^ Laboratory of Priority Terrestrial Vertebrates, Faculty of Biology Universidad Michoacana de San Nicolás de Hidalgo Morelia Mexico; ^3^ Instituto de Investigación en Recursos Cinegéticos (IREC), CSIC‐UCLM‐JCCM Ciudad Real Spain; ^4^ CEFE, Université Montpellier, CNRS, EPHE, IRD Montpellier France

**Keywords:** carnivore conservation, distance to the niche centroid, environmental suitability, Maxent, population monitoring

## Abstract

The distribution range and population abundance of species provide fundamental information on the species–habitat relationship required for management and conservation. Abundance inherently provides more information about the ecology of species than do occurrence data. However, information on abundance is scarce for most species, mainly at large spatial scales. The objective of this work was, therefore, to provide information regarding the population status of six wild felids inhabiting territories in Mexico that are inaccessible or politically unstable. This was done using species distribution models derived from occurrence data. We used distribution data at a continental scale for the wild felids inhabiting Mexico: jaguar (*Panthera onca*), bobcat (*Lynx rufus*), ocelot (*Leopardus pardalis*), cougar (*Puma concolor*), margay (*Leopardus wiedii*), and jaguarundi (*Herpailurus yagouaroundi*) to predict environmental suitability (estimated by both Maxent and the distance to niche centroid, DNC). Suitability was then examined by relating to a capture rate‐based index, in a well‐monitored area in central western Mexico in order to assess their performance as proxies of relative abundance. Our results indicate that the environmental suitability patterns predicted by both algorithms were comparable. However, the strength of the relationship between the suitability and relative abundance of local populations differed across species and between algorithms, with the bobcat and DNC, respectively, having the best fit, although the relationship was not consistent in all the models. This paper presents the potential of implementing species distribution models in order to predict the relative abundance of wild felids in Mexico and offers guidance for the proper interpretation of the relationship between suitability and population abundance. The results obtained provide a robust information base on which to outline specific conservation actions and on which to examine the potential status of endangered species inhabiting remote or politically unstable territories in which on‐field monitoring programs are not feasible.

## Graphical abstract

Distribution of felids population abundance in unstable territories.

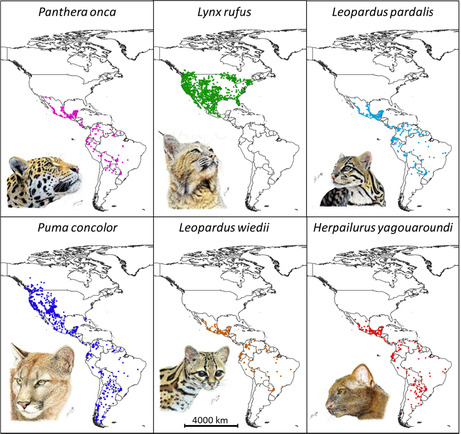

## 1 INTRODUCTION

Determining the distribution range and population abundance of wildlife and their spatiotemporal variation is a critical issue in applied ecology and conservation biology. Their spatial ranges provide important information on species–habitat relationships that is fundamental as regards supporting decision‐making in management and conservation (e.g., Baker et al., 2020). In this context, abundance data contain more information on population viability than do occurrence records (e.g., Ehrlén & Morris, 2015; Williams & Araújo, 2000), even when the underlying mechanism determining species occurrence is also based on population demography (e.g., Brown, 1984). However, obtaining precise information on population abundance such as relative/absolute abundance is more costly than recording occurrence data owing to the huge sampling effort required, especially when carried out on a large scale, which may be logistically and economically impossible (Pearce & Boyce, 2006). In certain circumstances, for example, the effort required to obtain abundance data concerning elusive species in inaccessible territories is, at a regional scale (e.g., >50,000 km^2^), simply unaffordable. Many researchers have consequently attempted to explore the relationship between species abundance and environmental suitability (in a broad sense), with the latter being derived from species distribution models (hereafter SDM) parameterized on highly accessible occurrence data (e.g., Carrascal et al., 2015; Yañez‐Arenas et al., 2012, 2014).

The relationship between environmental suitability and population abundance has been assessed for a wide variety of taxa, although a strong positive relationship has not always been evidenced (Dallas & Hastings, 2018; Lee‐Yaw et al., 2022, but see Weber et al., 2016). This suggests low generality and the a priori limited usefulness of environmental suitability as a predictor of spatial patterns of abundance. It has subsequently been argued that the weakness of this association is, in part, explained by a triangular rather than a linear relationship between suitability and abundance (VanDerWal et al., 2009), meaning that population abundance will usually be low in locations with low suitability, whereas abundance values can be either high or low in locations with high suitability (see also Jiménez‐Valverde et al., 2021). Environmental suitability has, therefore, been suggested as a proxy for the population abundance levels that local environmental conditions are able to support. This has opened up new possibilities as regards interpreting patterns of suitability as population‐related parameters (e.g., Acevedo et al., 2017; Muñoz et al., 2015; VanDerWal et al., 2009; but see Lee‐Yaw et al., 2022), although, again, wedge‐shaped relationships are not always detected (e.g., Yañez‐Arenas et al., 2012).

Both the challenges of predicting the distribution of abundance patterns by means of environmental suitability and the opportunities to do so increase when working with carnivores, especially wild felids, since obtaining presence and abundance data is particularly costly in the case of these species. Their ecological traits, which are related principally to low local population abundance (Olivero et al., 2016; Shrestha et al., 2014) imply low detection rates when conventional population monitoring methods are applied (Anile et al., 2012; Ceballos et al., 2010; Kilshaw et al., 2015). The resulting small sample sizes affect not only the availability of abundance data, but also the precision of the estimates obtained (Charre‐Medellín et al., 2021). In this study, we have obtained suitability predictions employing two different species distribution models algorithms for six wild felids: the jaguar (*Panthera onca*), bobcat or red lynx (*Lynx rufus*), ocelot (*Leopardus pardalis*), cougar (*Puma concolor*), margay (*Leopardus wiedii*), and jaguarundi (*Herpailurus yagouaroundi*). Suitability predictions were compared with relative abundance indices in an intensively surveyed area of central‐western region of Mexico, to examine the relationship between environmental suitability and population relative abundance. The results are not only meaningful for the management and conservation of these species in the area studied, but also provide the elements required to assess the potential status of endangered species in areas with logistical, economical, or security constraints (e.g., territories in which ongoing armed conflicts are taking place) where it is not possible to carry out field surveys.

## 2 MATERIALS AND METHODS

### 2.1 Study species and study area

Of the six felid species present in Mexico, the ocelot, jaguar, margay, and jaguarundi are mainly associated with tropical habitats, while the cougar and red lynx are mainly associated with temperate and/or arid habitats (Ceballos et al., 2010). All six species can be observed from sea level to above 3000 m.a.s.l.; however, most records of the four species of tropical affinity occur below 1000 m.a.s.l. (Ceballos et al., 2010). The home range of the six species is highly variable, from 10.5 km^2^ for a margay to over 300 km^2^ for jaguars and pumas (Konecny, 1989; Nuñez‐Perez & Miller, 2019). Of the six felid species, the margay has mainly arboreal habits (Monterrubio‐Rico et al., 2023).

The main study area was the central‐western region of Mexico, which includes Jalisco, Colima, Michoacán, Guerrero, and the State of Mexico, with an area of 228,776 km^2^ (11.65% of Mexico area). The topography is complex owing to the convergence of the Sierra Madre Occidental, the Sierra Madre del Sur, and the Trans‐Mexican Volcanic Belt, three of the four most rouged mountain ranges in the country. Moreover, the lower Balsas River basin is located between them. This geographic scenario has broad ecological gradients that contain the majority of the vegetation types found in Mexico (Pronatura México AC & The Nature Conservancy, 2007). This heterogeneous topography includes 11 different ecoregions, with the predominant types of natural vegetation in the study area being tropical and temperate forests and scrublands (Olson et al., 2001). The landscapes have multiple ravines and canyons with vigorous riparian vegetation cover that may constitute dispersal corridors among populations in the North, South and central Mexican Pacific. The region is, therefore, considered a priority for the conservation of many species for all of the aforementioned reasons (CONANP, 2009; Núñez, 2012; Rabinowitz & Zeller, 2010; Rodríguez‐Soto et al., 2011, 2013). Please note that populations of the six aforementioned wild feline species still exist in Mexico (Pronatura México AC & The Nature Conservancy, 2007).

### 2.2 Distribution data

The SDMs were generated by collecting species presence records throughout their entire distribution range (continental scale) in order to incorporate the totality of the environmental tolerance limits of each species into the model (Sánchez‐Fernández et al., 2011). Several national and international databases were revised, including the World Network of Information on Biodiversity REMIB (http://www.conabio.gob.mx/remib/doctos/remib_esp.html), the Biodiversity Informatics Unit, UNIBIO (http://unibio.unam.mx/), the Global Biodiversity Information Facility, GBIF (http://www.gbif.org/), and the Mammal Networked Information System, MaNIS (http://manisnet.org/). A total of 8700 records concerning the six species were obtained for their entire distribution range in America. Both the common and the scientific names of the species jaguar (*Panthera onca*), bobcat or red lynx (*Lynx rufus*), ocelot (*Leopardus pardalis*), cougar (*Puma concolor*), margay (*Leopardus wiedii*), and jaguarundi (*Herpailurus yagouaroundi*) were used as search criteria. The results obtained were then filtered in order to limit the search to the American continent. All records went through a cleaning process in which some records were excluded by considering the following criteria: records outside the recognized distribution range (IUCN range maps), records outside the continental margin, records located within urban areas (zoos), and fossil records. The set of records selected was then transferred to a grid of 10 × 10 km squares, which was used as the territorial unit for the SDMs so that there could only be one record of the same species in the same grid. A total of 4508 square cells with species presence were subsequently used to generate the SDMs, distributed as follows: bobcat (*n* = 2268), cougar (*n* = 1091), ocelot (*n* = 345), jaguar (*n* = 298), jaguarundi (*n* = 286), and margay (*n* = 220).

### 2.3 Species distribution modeling

**FIGURE A1:**
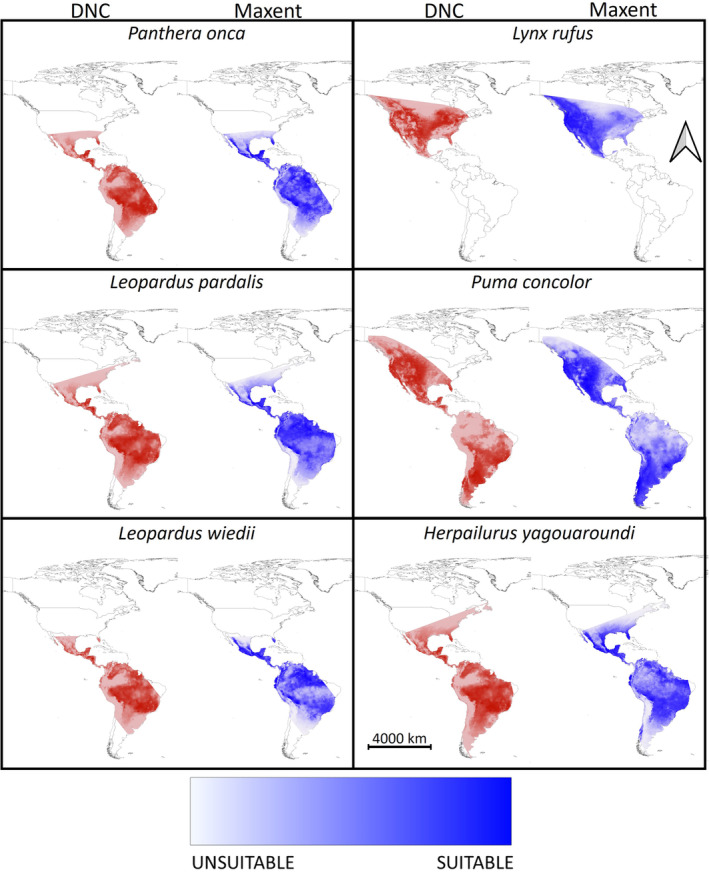
Average model distribution obtained for each species for the TSA with two algorithms: Maxent and distance to niche centroid (DNC). Darkest colors in the prediction indicate higher suitability.

Recent studies have shown that the area used during the SDM process has a dominant influence on the outcome of the model, thus highlighting the importance of considering the accessible area of the species (e.g., Acevedo et al., 2017; Barve et al., 2011). We accordingly used the procedure proposed by Acevedo et al. (2012), which is based on the third‐degree polynomial of latitude (lat) and longitude (long; TSA; Trend Surface Analysis), to delimit the specific geographic area to be considered for the SDMs (Figure A1 in Appendix A). Briefly, this consists of a logistic model for each species at the continental scale (10 × 10 km^2^), in which TSA (lat, long, lat*long, long*lat^2^, long^2^*lat, lat^2^, long^2^, lat^3^, and long^3^) are used as the predictor variables. The predictions provided by the model are then used to define the area for environmental modeling, selecting squares with a prediction higher than the minimum predicted value for a presence.

Using the TSA‐delimited area for each species, 11 climatic variables, obtained from WorldClim v2 (period 1970–2000), were employed as predictors of environmental suitability for each species (see Table 1; Fick & Hijmans, 2017). Quarterly variables were not used since differences in quarterly aggregation led to sharp contrasts between nearby cells (Bede‐Fazekas & Somodi, 2020). To avoid collinearity, VIF has been employed for each species in a first step, with the “vifstep” function of the “usdm” package (Naimi et al., 2014). Using the presence records of the six feline species and the climatic variables selected as a basis, two algorithms were employed in order to predict environmental suitability: Maxent (Phillips et al., 2006) and distance to niche centroid (DNC; Maguire Jr., 1973). These algorithms were selected because they have been consistently used in previous studies and are considered an adequate means to infer patterns of species abundance (e.g., Altamiranda‐Saavedra et al., 2020; Osorio‐Olvera, Yañez‐Arenas, et al., 2020; Yañez‐Arenas et al., 2014).

**TABLE 1:** Eleven climatic variables used as predictors in the species distribution models. These variables were obtained from WorldClim v2 (Fick & Hijmans, 2017).

Code	Description
BIO1	Annual mean temperature
BIO2	Mean diurnal range (mean of monthly (max temp – min temp))
BIO3	Isothermality
BIO4	Temperature seasonality
BIO5	Maximum temperature of warmest month
BIO6	Minimum temperature of coldest month
BIO7	Temperature annual range
BIO12	Annual precipitation
BIO13	Precipitation of wettest month
BIO14	Precipitation of driest month
BIO15	Precipitation seasonality

**FIGURE 1:**
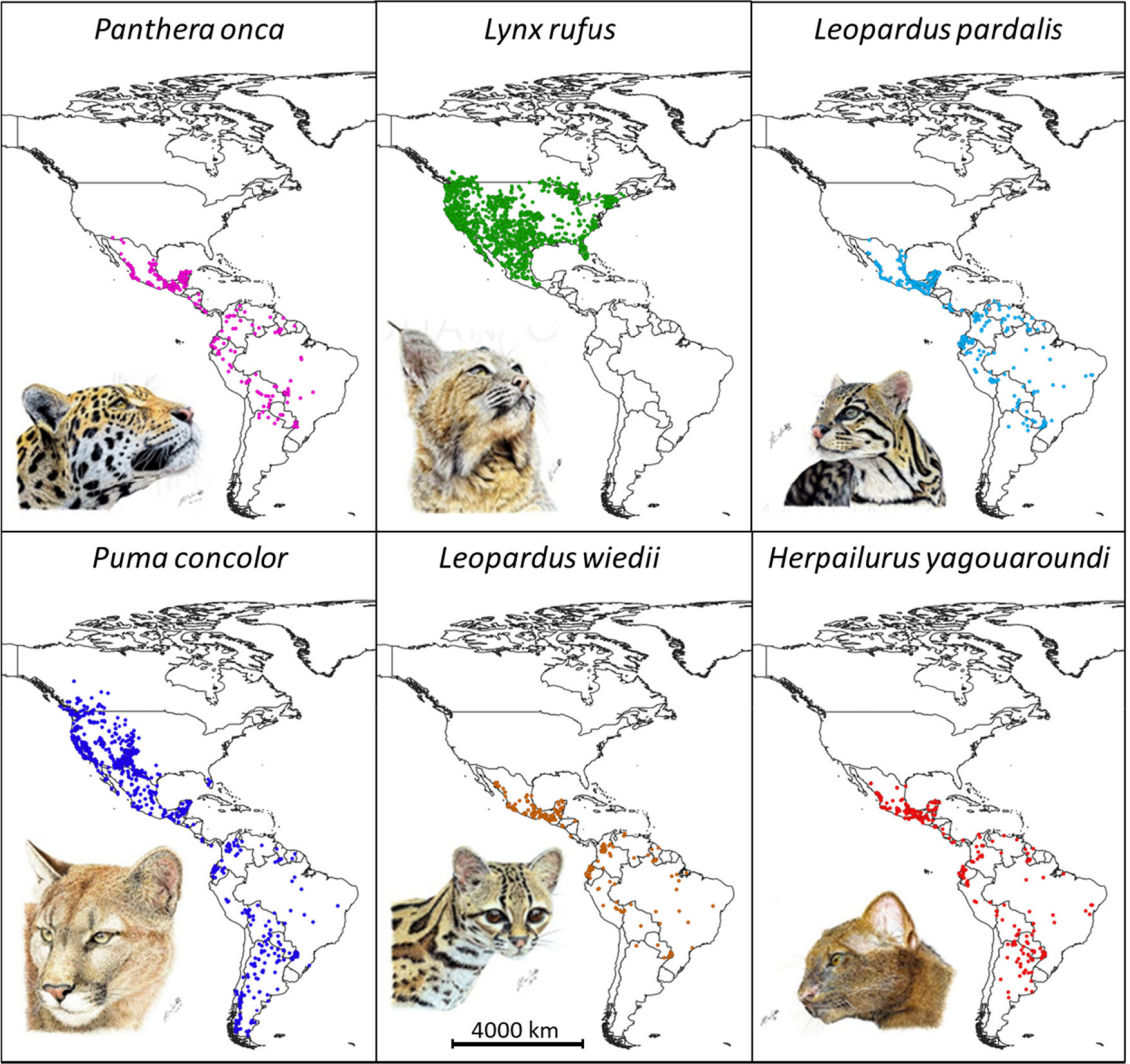
Occurrences used to parameterize species distribution models. See text for data sources. Illustrations by Juan Carlos Martínez.

Prior to environmental modeling, we divided the occurrence dataset into training (70% of locations) and test datasets (30%). This procedure was repeated 10 times in order to obtain averaged models for both Maxent and DNC (replicates). The models were parameterized on the training datasets (Figure 1). In the case of Maxent, we extracted the environmental values of presences and 10,000 background points. We used a Human Influence Index layer (The Last of the Wild Project version 2 http://sedac.ciesin.columbia.edu/data/collection/wildareas‐v2) as a bias layer to distribute the background points according to human influence, thus allowing us to control for sampling bias in the presence records (Fourcade et al., 2014; Tellería et al., 2014). With regard to parameterization, the “create response curves,” “make pictures of predictions,” and “do jackknife test of variable importance” module and options were performed. We used the output format Cloglog (Phillips et al., 2017). Only the “linear,” “quadratic,” and “product” (i.e., pair‐wise interactions between predictors) features were utilized in order to avoid overfitting models, and specially in too complex and unrealistic response curves (Elith et al., 2011; Low et al., 2021; Merow et al., 2013). Analyses were carried out using Maxent version 3.4.4.

In the DNC analysis, we used only the presence records to extract environmental values for each location. In order to reduce the number of predictor variables and ensure independence between them (a part of VIF analyses), we assessed predictors of collinearity in the presence dataset and removed those that were redundant with the “correlation_finder” function of the “ntbox” R package (a Spearman correlation higher than |0.8|; Osorio‐Olvera, Lira‐Noriega, et al., 2020). The niche centroid was then calculated for each species from the mean of each predictor in locations with a presence with the “mean” function, and the Mahalanobis distance to the niche centroid was calculated for each location with the “mahalanobis” function of the “stats” R package (Osorio‐Olvera et al., 2019; Soberón et al., 2018). All analyses were performed using R v. 4.1.0 (R‐Core Team, 2021).

The area under the receiver operating characteristic curve (AUC) was used to assess the discriminatory capacity of the models for the test datasets (Fielding & Bell, 1997; Lobo et al., 2008). Model predictions (from Maxent and DNC) for each species were then projected onto central western Mexico (Jalisco, Colima, Michoacán, Guerrero, and the State of Mexico) at a resolution of 1 km^2^, which allowed us to obtain an averaged model from 10 replicas. The agreement between the Maxent and DNC model predictions was estimated using Cohen's weighted kappa coefficient (Cohen, 1960). The weighted kappa is a modification of Cohen's kappa that considers the closeness of agreement between categories when there are more than two, penalizing disagreement more severely when the difference between categories is greater. The predicted values were then divided into four quantile‐based categories for comparison. The weighted kappa index ranged from −1 (complete disagreement) to 1 (complete agreement), with a value of 0 indicating a concordance equivalent to that expected by chance.

### 2.4 Relative abundance data and their relationship with environmental suitability

**FIGURE A2:**
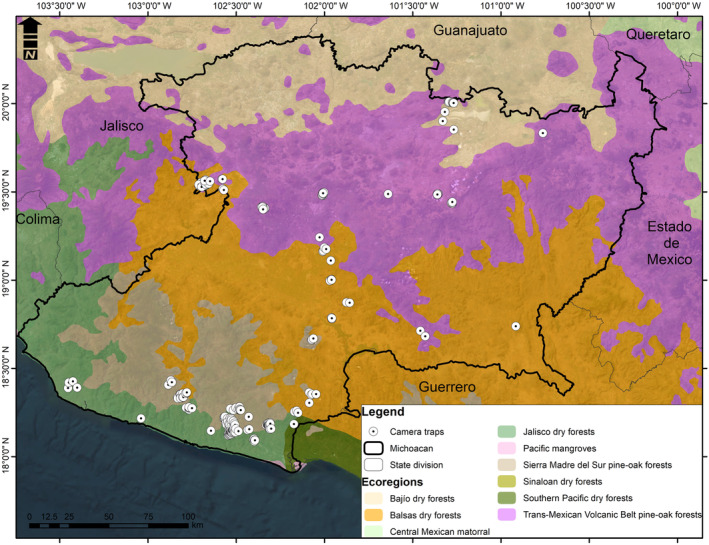
Location of camera traps in Michoacán, Mexico (2007–2019). Ecoregions taken from Olson et al. (2001).

Between 2008 and 2020, 241 sites were sampled with camera traps in order to estimate the relative abundance (capture rate) of wild felids (Figure A2 in Appendix A). The number of trapping sites, ecoregions sampled, and sampling periods varied over time (Table A1 in Appendix A). The sites were selected on the basis of geographic accessibility, vegetation type, security of access, and support from local people.

The camera brands were Stealth Cam Skout 7MP, Wildview MP Digital Scouting Cam, and Bushnell 8MP Trophy Cam Trail. Each device was programmed to take three photographs per capture event and to reactivate for the next capture event 1 min later. No baits or olfactory attractants were used so as to ensure that only the natural movements of the animals were recorded (Long et al., 2012). The camera traps were placed on the edges of trails, paths, and springs in order to maximize the probability of recording the presence of felines (Silver et al., 2004). The relative abundance of each species was estimated from the number of independent events in the 1 km^2^ square grid divided by the total number of days that camera traps were operating in each square. Independent events were defined as those obtained for the same species and camera‐trap station, but taken at least 24 h apart, or when more than one individual was recorded in a photograph (e.g., Wearn & Glover‐Kapfer, 2017).

The relationships between capture rates and predicted suitability at square‐level were assessed using quantile regression (VanderWal et al., 2009). This type of regression was employed because it is appropriate for dealing with unequal variance in the distribution of *Y* along *X*, since it is able to estimate rates of change in all parts of the distribution of the response variable (Cade & Noon, 2003). The fit of this type of regression was checked by taking into account the slope (percentile) and intercept of the function line, along with the mean goodness‐of‐fit measure for quantile regression for different percentiles (R1), which is the weighted sum of absolute residuals (Koenker & Machado, 1999; see also Jiménez‐Valverde, 2011 for further explanations about R1). In our case, linear quantile regressions were fitted to the 60th, 70th, 80th, 85th, 90th, 91st, 93rd, 95th, 97th, and 99th percentiles, and the rates of change between them were estimated in order to provide a complete picture of the relationship between relative abundance and suitability and to determine which of them provided the best upper bound of the response (i.e., a higher R1 and slope and an intercept close to 0; see Muñoz et al., 2015). We developed quantile regressions for each species and both SDM algorithms (Maxent and DNC). Analyses were performed using the “quantreg” R package version 5.85 (Koenker, 2021).

## 3 RESULTS

**FIGURE 2:**
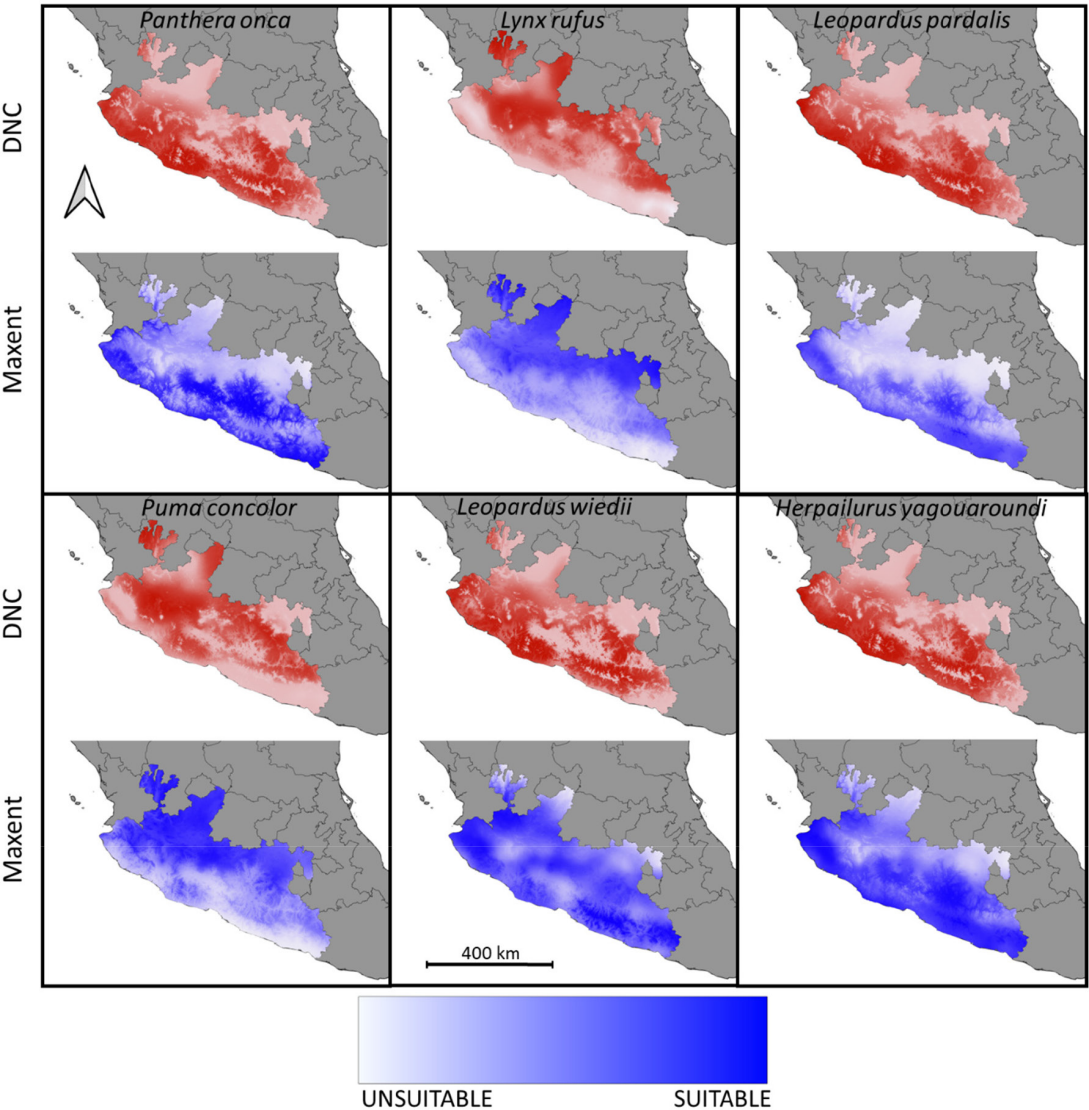
Distribution models obtained for each species with two algorithms, downscaled to 1 × 1 km squares (see Table A2 in Appendix A): Maxent (blue ramp) and distance to niche centroid (DNC; red ramp), at the study region level, central western Mexico. Darkest colors of the prediction indicate higher suitability.

Species' response to environmental gradients according to Maxent and DNC are shown in Table A2 in Appendix A. According to the internal validation, Maxent generally achieved a higher predictive performance (AUC values) than DNC for the test datasets (Table 2). Nevertheless, similar patterns were observed with both SDM algorithms, although with disparities in certain areas (Figure A1 in Appendix A), even when were downscaled to the study area (Figure 2). The equivalence of the environmental suitability patterns predicted by the two algorithms was confirmed by Cohen's weighted kappa coefficient (Table 3).

**TABLE 2:** Internal validation of the species distribution models for the six feline species in the test (independent) datasets for the Maxent and distance to niche centroid (DNC) models.

Species	Area under the ROC curve (SD)	
	Maxent	DNC
Jaguar	0.797 (0.017)	0.628 (0.025)
Bobcat	0.649 (0.005)	0.675 (0.006)
Ocelot	0.775 (0.014)	0.589 (0.024)
Cougar	0.779 (0.012)	0.699 (0.014)
Margay	0.787 (0.021)	0.514 (0.024)
Jaguarundi	0.797 (0.026)	0.612 (0.019)

**TABLE 3:** Estimation of the agreement between predicted patterns of environmental suitability from the two algorithms tested: Maxent and distance to niche centroid.

Species	Cohen's weighted kappa coefficient (CI 95%)
Jaguar	0.40 (0.40–0.40)
Bobcat	0.72 (0.72–0.73)
Ocelot	0.66 (0.65–0.66)
Cougar	0.44 (0.44–0.44)
Margay	0.33 (0.33–0.34)
Jaguarundi	0.45 (0.45–0.46)

*Note*: This analysis of the six feline species was spatially limited to the study area, the central‐western region of Mexico. Cohen's weighted kappa coefficient was used.

Jaguar, jaguarundi, margay, and ocelot showed an affinity for tropical climate zones, since the areas with the highest suitability scores were in the Pacific coast region (Figure 2). In the case of the bobcat, however, suitability was associated more with the Trans‐Mexico volcanic belt, the upper parts of the Sierra Madre del Sur and the lower Balsas basin, while the cougar obtained a diffuse distribution pattern throughout the study region (Figure 2).

With a cumulative sampling effort of 26,784 camera‐trap days, wild felids were recorded on 241 sampling sites, jaguars on 17, bobcats on 25, ocelots on 85, cougars on 119, margays on 52, and jaguarundis on 16 sites. During the course of the sampling period, 1688 independent events of wild felids were obtained (jaguar = 60, bobcat = 560, ocelot = 507, cougar = 402, margay = 134, jaguarundi = 25).

**FIGURE A3:**
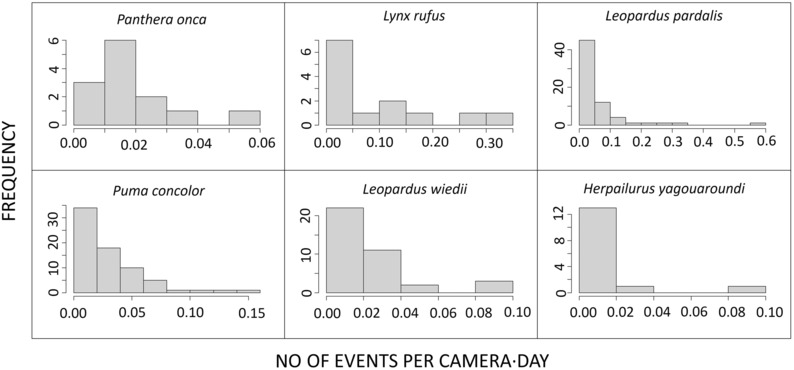
Distribution of the different relative abundance values (no. of events per camera per day) for six species obtained with camera traps.

Of the 146 squares for which relative abundance data were available, the species detected in the largest number of squares was the cougar, in a total of 71 squares with counts, with a maximum and a mean of 0.143 and 0.031 events per camera day, respectively. This was followed by the ocelot in 66 squares, with a maximum of 0.563 events and a mean of 0.058, the margay, in 38 squares, with a maximum of 0.100 and a mean of 0.022, and the jaguarundi in 15 squares, with a maximum of 0.100 and a mean of 0.013 events, all per camera day. The jaguar and lynx had the lowest number of squares with data (13), with a maximum of 0.059 and 0.325, and a mean of 0.020 and 0.088 events per camera day, respectively. Figure A3 in Appendix A shows the distribution of the relative abundance values of the different species.

**FIGURE 3:**
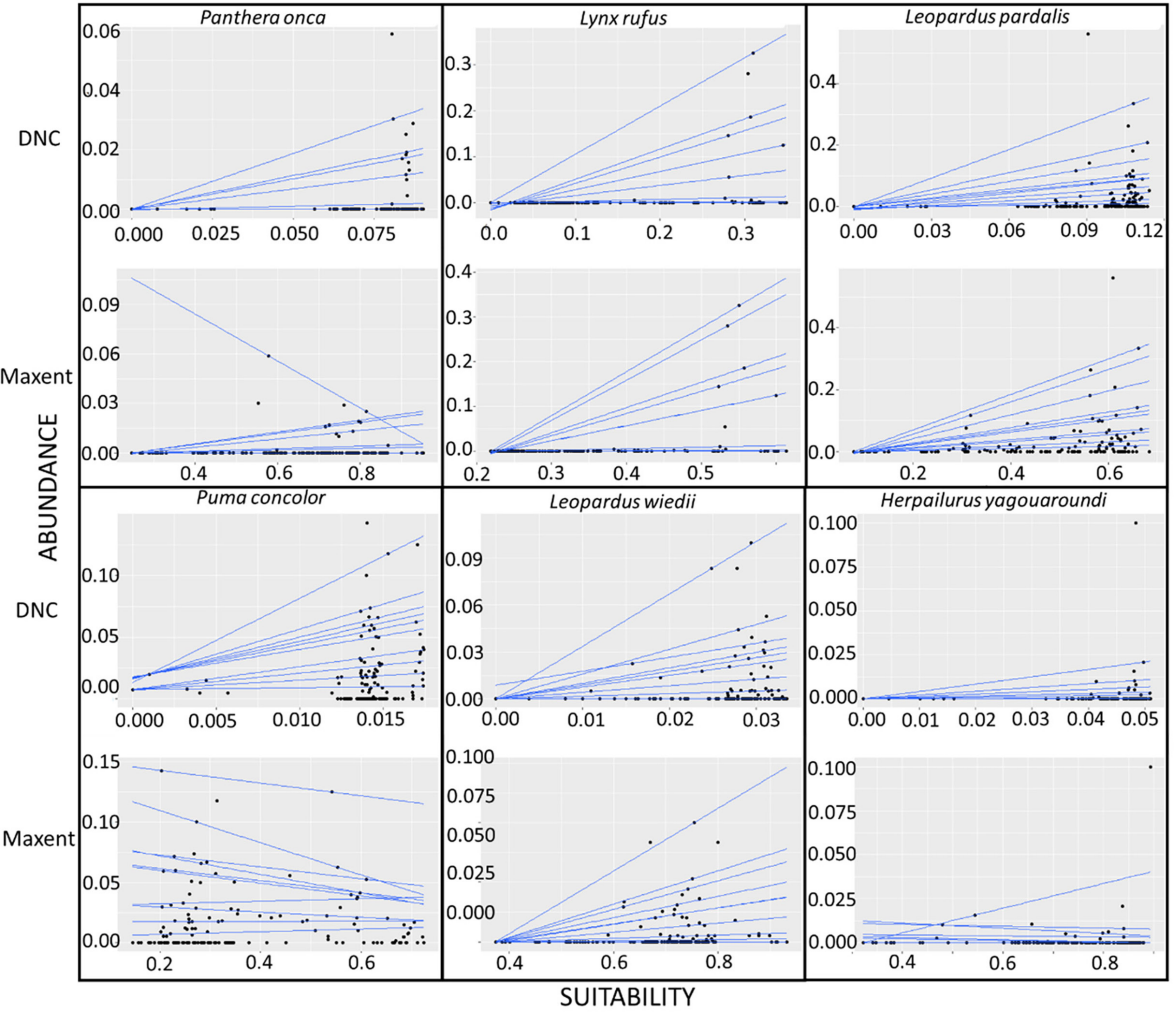
Relationship between observed abundance and environmental suitability predicted by Maxent and distance to niche centroid (DNC). Quantile regressions were fitted for 60th, 70th, 80th, 85th, 90th, 91st, 93rd, 95th, 97th, and 99th percentiles.

The relation between the environmental suitability predicted by the different approaches and the observed relative abundance values had R1 values of between 0.001 and 0.509, slope values of between 0.010 and 4.063, and intercept values of between −0.128 and 0.030. Upon observing these three components, it will be noted that the species with the best fit is the bobcat (i.e., a higher R1 and slope, and an intercept close to 0) in both the Maxent and DNC models (Table 4 and Figure 3). However, several species, such as the jaguar and the cougar, had a worse fit in one or both of the SDM algorithms (Table 4 and Figure 3). The DNC predictions had a better fit to observed abundances than Maxent predictions (Figure 3 and Table 4).

**TABLE 4:** Statistical parameters of quantile regressions between environmental suitability predicted by the Maxent and distance to niche centroid (DNC) models and the observed abundances for the six feline species in the state of Michoacán.

Species	Algorithm	Parameters			Selected quantile
		R1	Slope	Intercept	
Jaguar	Maxent	0.074	0.033	−0.008	95th
	DNC	0.039	0.226	0.000	97th
Bobcat	Maxent	0.408	0.564	−0.128	95th
	DNC	0.509	1.050	0.000	99th
Ocelot	Maxent	0.140	0.386	−0.036	95th
	DNC	0.058	1.841	0.000	97th
Cougar	Maxent	0.001	0.010	0.030	85th
	DNC	0.037	4.063	0.016	97th
Margay	Maxent	0.065	0. 120	−0.045	95th
	DNC	0.031	3.357	0.000	99th
Jaguarundi	Maxent	0.064	0.070	−0.022	99th
	DNC	0.023	0.418	0.000	99th

## 4 DISCUSSION

The basic parameter required for the conservation and management of wildlife populations is their population size. Central western Mexico is an important region for the conservation of wild felids owing to its biogeographical characteristics, but many national territories are, unfortunately, not accessible for research. It is, therefore, essential to assess species distribution models in order to provide information regarding the distribution and relative abundance of species in order to design local actions for wildlife conservation, mainly in remote and/or inaccessible areas. Our results indicated differences between both approaches and species, showing: (i) a better performance of the DNC approach as regards predicting potential abundance, and (ii) absence of general patterns, and that (iii) species' characteristics and/or local factors could blur the relationship between abundance and environmental suitability. In general, our results suggest that SDM could be a feasible option with which to obtain information on species relative abundance, but further research should be carried out to describe the analytical procedures and the requirements of the abundance data so as to obtain consistent proxies of relative abundance from SDM.

### 4.1 On the capture rate as a relative abundance index

The conservation of wild felids depends on reliable estimates of their populations at both global and regional scales, which can, in practice, be impossible or very costly to obtain, particularly in the case of inaccessible regions (Maffei et al., 2004; Trolliet et al., 2014). In this study, the camera traps allowed the estimation of a proxy of population size through the estimation of the capture rate, expressed as the number of independent events recorded for a species in relation to the sampling effort, usually measured in terms of camera days (Carbone et al., 2001; Lijun et al., 2019).

Indices of relative abundance assume that the capture rate varies in a constant and linear manner with the real abundance of the species (Pollock et al., 2002). This linear relationship has been documented for a large number of species, including felids and their potential prey (Carbone et al., 2001; Palmer et al., 2018; Rovero & Marshall, 2009). However, the biases that these simple indices may have by not considering the temporal and spatial variation in species detection have also been documented in recent years (Kolowski et al., 2021; Sollmann et al., 2013). Given the strong assumption that it is necessary to use an index and that it is not easily tested, indices should, therefore, be avoided unless no reasonable alternative is possible. In this respect, O'Brien (2011) described that indices are typically used when: (i) the target species is difficult to capture or observe, (ii) it is logistically difficult or too expensive to implement surveys that are capable of determining detection probability, or (iii) there is historical precedent for index surveys. Wild felids inhabiting inaccessible areas comply with all of these circumstances. Since we are aware of their limitations when interpreting the results, we consider that the use of the capture rate from camera traps as a proxy of relative abundance of wild felids for the study area is a reasonable method.

### 4.2 Distribution of species abundance

Central‐western region of Mexico has been considered an important region for the conservation of wild felids (Rodríguez‐Soto et al., 2011). The results reported here are important, not only for the management and conservation of these species in the study area, but also as regards providing (predicted) data for territories that are politically or socially unstable at the present time, and in which it is impossible or very difficult to implement field monitoring programs (Carpio‐Domínguez, 2021).

Unfortunately, some regions in Mexico have become inaccessible for research, and particularly for wildlife monitoring, owing to the insecurity caused by criminal groups that are taking over remote territories, including natural protected areas (Carpio‐Domínguez, 2021). One of these is the study area (comprising Jalisco, Colima, Michoacán, and Guerrero) which, despite having a large number of protected natural areas, is also one of the most insecure regions in Mexico. Given this situation, conservation efforts based on the ability to predict potential abundances by means of field surveys that allow the management and conservation of endangered species such as wild felids have become very important.

One of the key parameters for the management and conservation of wild felid populations and communities is that of discovering the abundance of the species, which can be expressed in absolute and/or relative terms (Hopkins & Kennedy, 2004). There has consequently been an attempt to relate species abundance to characteristics associated with their geographic and/or environmental distribution. This ranges from the “center‐abundant” hypothesis, which assumes that species are more abundant toward the center of their geographic distribution (Brown, 1984), to predictors that use species distribution models as an approximation with which to estimate potential abundances (see also Tôrres et al., 2012; VanderWal et al., 2009). However, it has recently been proposed that the abundance of a species on a given site is related to the position of the site in the fundamental niche of the species (see also, Osorio‐Olvera, Yañez‐Arenas, et al., 2020). This relationship is known as the distance to niche centroid (DNC; Dallas & Hastings, 2018). It is for this reason that we have, in this work, employed the two most commonly used approaches to estimate potential: Maxent and DNC. Large spatial scale approaches are promising tools with which to generate coarse information on species abundance in territories in which field‐based methods are not an alternative. The predicted relative abundances are valuable as regards designing actions with which to monitor populations and communities with the purpose of implementing effective conservation programs.

### 4.3 Different performance of algorithms

There were certain differences between the algorithms when assessed using independent distribution data, since the internal validation was generally higher in Maxent (Table 2). One of the possible explanations for this is that relationships modeled between environmental conditions and occurrence using different algorithms may yield similar patterns on a coarse scale, but have substantial differences at the local scale (Guisan & Zimmermann, 2000; Tôrres et al., 2012). However, while Maxent performed better than DNC when assessed with distribution data, the situation was the other way round when assessed with relative abundance data (see below). This suggests that the discriminatory capacity of SDM does not always correlate to the strength of the relationship between suitability and abundance (but see Jiménez‐Valverde et al., 2021).

The best fit between felid relative abundance and habitat suitability was generally observed using DNC when compared to Maxent. This is consistent with previous findings, which have shown that population approximations are better when using DNC than with any other method (Osorio‐Olvera et al., 2019). In their work, Pérez‐Irineo et al. (2019) evaluated the relationship between the suitability and population (density) of four felid species (*L. rufus*, *L. pardalis*, *P. concolor*, and *P. onca*) using Maxent and DNC as approximations, and a better fit was obtained with DNC, as occurs in our work. The results of this study suggest that the relationship between the relative abundance of felids and the environment may not follow a generalized pattern. This has been observed in other studies that have evaluated this relationship for a suite of species, especially birds and mammals (Osorio‐Olvera, Yañez‐Arenas, et al., 2020; Santini et al., 2019). It is possible that factors associated with species biology (dispersal ability, availability of prey and presence of competitors and/or predators) and disturbance factors at the local level affect the relationship between relative abundance and environmental suitability (de la Fuente et al., 2021; Osorio‐Olvera et al., 2016; Pérez‐Irineo et al., 2019). We, therefore, suggest the need to continue evaluating the relationship between species abundance and environmental suitability at the local or regional level, incorporating variables associated with the characteristics of the region (biotic and abiotic).

### 4.4 Relevance of abundance data when assessing occurrence–abundance relationship

The performance of different SDM algorithms when predicting abundance has already been discussed in various studies (Martínez‐Meyer et al., 2013; Osorio‐Olvera et al., 2019; Osorio‐Olvera, Yañez‐Arenas, et al., 2020; Yañez‐Arenas et al., 2014), and the inconsistent performance of Maxent has been explained with regard to whether its output is (or is not) truly suitable and to its capability to predict actual (rather than potential) distribution (Yañez‐Arenas et al., 2014; see also Osorio‐Olvera et al., 2019). Our results do not provide a directional hypothesis in this respect. In the context of the data considered in this study, Maxent performed as well as DNC for species such as the bobcat, margay, and ocelot, but predicted low suitability for locations in which the observed abundance of the species was actually high (Figure 3). A number of observations are necessary here. The absence of a clear relationship between suitability and abundance when using field data does not always mean that it does not exist, but that proper data with which to assess it are not available. Although many studies have focused on the effects of the quality of occurrence data (and species range) and predictors in this relationship (e.g., Jiménez‐Valverde et al., 2021; Yañez‐Arenas et al., 2014), the effect of bias and the representativeness of abundance data have largely been ignored.

For a proper assessment of the relationship between suitability and abundance, reliable indices of environmental suitability are required, as is species abundance data, which would ideally: (i) be obtained using reliable methods, (ii) be distributed across the whole suitability range, and (iii) reach maximum abundance values, at least under the environmental conditions of the area in which the relationship is evaluated. In the majority of cases, however, observed abundance values greater than zero are clustered only in part of the suitability range (Figure 3; see also Martínez‐Gutiérrez et al., 2018), and/or those locations in which species abundance is highest are not considered (but see Muñoz et al., 2015). Inadequate abundance data can, therefore, lead to a poor fit in the relationship (Yañez‐Arenas et al., 2014), which is often interpreted as the inability of SDM to predict abundances (e.g., Dallas et al., 2017). Here, we therefore emphasize that the characteristics of abundance data should be critically evaluated when assessing the relationship between environmental suitability and local population abundance. These observations show that the relationship among environmental suitability, species distribution and population abundance is complex and that many factors should be considered before it can be assessed (Bohner & Diez, 2020; Holt, 2020).

## AUTHOR CONTRIBUTIONS

**Juan F. Charre‐Medellín:** Conceptualization (equal); data curation (equal); formal analysis (equal); investigation (equal); writing – original draft (equal); writing – review and editing (equal). **David Ferrer‐Ferrando:** Conceptualization (equal); data curation (equal); formal analysis (equal); investigation (equal); writing – original draft (equal); writing – review and editing (equal). **Tiberio C. Monterrubio‐Rico:** Supervision (equal); writing – original draft (equal); writing – review and editing (equal). **Javier Fernández‐López:** Conceptualization (equal); formal analysis (equal); investigation (equal); writing – original draft (equal); writing – review and editing (equal). **Pelayo Acevedo:** Conceptualization (equal); data curation (equal); formal analysis (equal); investigation (equal); supervision (equal); writing – original draft (equal); writing – review and editing (equal).

## FUNDING INFORMATION

We would like to thank the Coordinación para la Investigación Científica at UMSNH for partially funding this research, and CONACYT for the postgraduate studies fellowship awarded to JFCM (239248). JFCM is grateful to the General Office of Academic Affairs, UNAM and the Environmental Geography Research Centre, UNAM for the postdoctoral grant. DF received support from the UCLM through a predoctoral scholarship (2020‐PREDUCLM‐16022). JFL was funded by the Margarita Salas grant from the European Union—Next Generation EU through the Complutense University of Madrid.

## CONFLICT OF INTEREST STATEMENT

The authors declare no conflict of interest.

## Data Availability

The dataset used in this study is publicly available in Zenodo Repository (https://zenodo.org/badge/DOI/10.5281/zenodo.8297706.svg).

## APPENDIX A

**TABLE A1:** Description table of the camera trap disposition for each region in the study area.

Ecoregions	Trap sites	Sampling effort (days)	Years
Bajio	8	88	2010–2011
Trans‐Mexican Volcanic Belt	55	9861	2010–2020
Balsas Basin	53	7431	2012–2018
Sierra Madre del Sur	63	4489	2008–2015
Coast	62	4915	2008–2015
Total	241	26,784	

**TABLE A2:** Percentage contribution values for each variable in the Maxent algorithm; mean and SD in the distance to niche centroid algorithm, obtained with the data of the six felids.

Algorithms	Percentage contribution of the variables (%)						
Maxent	Bio 1	Bio 2	Bio 3	Bio 4	Bio 13	Bio 14	Bio 15
Jaguar	44	3.4	7.7	‐	16.3	6.7	21.9
Bobcat	16.8	1.1	‐	46.4	1.6	23.5	10.7
Ocelot	35.3	5.2	6.5	‐	21.6	10	21.4
Cougar	24.2	36.7	26.9	‐	3.3	1.9	7
Margay	6	2.8	3.3	15.2	27.9	12.2	32.6
Jaguarundi	19	2.5	10.2	‐	31.2	4	33.1

Distance to Niche Centroid	Mean ± (SD)						
Jaguar	0.402 (0.036)	−0.207 (0.043)	0.066 (0.011)	‐	0.472 (0.041)	−0.081 (0.037)	0.337 (0.029)
Bobcat	0.330 (0.008)	0.185 (0.010)	‐	−0.476 (0.011)	0.151 (0.085)	−0.221 (0.014)	0.233 (0.019)
Ocelot	0.507 (0.019)	−0.475 (0.037)	0.345 (0.022)	‐	0.345 (0.032)	0.057 (0.033)	‐
Cougar	−0.248 (0.013)	0.602 (0.017)	−0.216 (0.011)	‐	−0.237 (0.012)	−0.186 (0.011)	0.185 (0.021)
Margay	0.020 (0.043)	−0.134 (0.046)	0.111 (0.028)	‐	0.460 (0.049)	−0.037 (0.021)	‐
Jaguarundi	0.480 (0.022)	−0.141 (0.026)	0.231 (0.026)	‐	0.554 (0.036)	−0.175 (0.030)	‐
